# Ventriculoperitoneal Shunt and Gastrostomy Tube Placement and Timing: A Database Analysis

**DOI:** 10.7759/cureus.23776

**Published:** 2022-04-03

**Authors:** David R Hallan, Elias Rizk

**Affiliations:** 1 Neurosurgery, Penn State Health Milton S. Hershey Medical Center, Hershey, USA

**Keywords:** mortality, revision, meningitis, infection, outcomes, g-tube, peg, shunt, neurosurgery

## Abstract

Background

Debate exists about the safety of ventriculoperitoneal shunt placement in the presence of a gastrostomy tube and the timing of these procedures from each other. Using a large database, we sought to determine the rates of shunt infection and revision in patients who had both devices placed, based on the timing between procedures.

Methods

We performed a retrospective database analysis using a multi-institutional database (TriNetX), looking at all patients diagnosed with gastrostomy tube with subsequent ventriculoperitoneal shunt placement and vice-versa. We also evaluated patients who had gastrostomy tubes and shunts placed at the same time. We categorized cohorts into patients with device placement after 1-10 days, 11-30 days, and after one month of the other. Our primary endpoints were shunt infection and shunt revision.

Results

Patients who had same-day gastrostomy tube and shunt placement had a shunt infection rate of 10.06% within five years, and 14.53% had a shunt revision. With prior shunting and subsequent gastrostomy tube placement within 1-10 days, 12.18% had shunt infections, and 17.88% had shunt revisions; for those who had subsequent gastrostomy tube placement within 11-30 days, shunt infections were seen in 10.57%, and shunt revisions in 19.41%; gastrostomy tube placement after one month or longer of shunt placement resulted in 15.39% of patients having shunt infections and 17.73% with shunt revision. Prior gastrostomy tube patients with subsequent shunt placement, within 1-10 days had shunt infection rates of 8.27% and revision rates of 14.39%; for shunt placement within 11-30 days, shunt infections were seen in 10.82%, and shunt revisions were done in 14.33% of patients; for shunt placement after one month or longer, shunt infection rate was 11.68%, and revision rate was 16.80%.

Conclusions

Our results demonstrate no significant difference in shunt infection rates and shunt revision rates between same-day gastrostomy tube and shunt placement versus placement within 1-10 days, 11-30 days, or any time after one month from one another.

## Introduction

Patients with neurologic injury requiring a ventriculoperitoneal shunt (VPS) often also require gastrostomy tube (G-tube) placement for nutritional support. Likewise, patients with neurologic injury requiring a G-tube for nutritional support may also have hydrocephalus requiring a VPS [1]. However, there is debate about the safety of ventriculoperitoneal shunt placement in the presence of a gastrostomy tube, and vice-versa, and the timing of such procedures from each other. The discourse stands on the risk of shunt malfunction and shunt infection with bacterial pathogens related to a G-tube [1-6]. Therefore, we sought to determine the shunt infection rates and shunt revision in patients who had both devices placed, based on the timing between procedures, using a large database. 

## Materials and methods

This was a retrospective comparative case-control study. We used a de-identified database network (TriNetX) to retrospectively query via ICD-10 and current procedural terminology codes to evaluate all patients with a prior G-tube placement with subsequent VPS placement, as well as patients with a prior VPS with subsequent G-tube placement. We also evaluated patients who had G-tubes and VPS placed at the same time. We categorized placement into cohorts for those patients who had device placement after 1-10 days, 11-30 days, and after one month of the other. Data were obtained from 62 health care organizations (HCOs) spanning 11 countries. The database includes variables on demographics, diagnoses, medications, laboratory values, genomics, and procedures. The identity of the HCOs and patients is not disclosed to comply with ethical guidelines regarding data re-identification. Because of the database's federated nature, an IRB waiver has been granted. Our use of this database and its validity has been disclosed by previous literature, and exact details of the network have been previously described [7-10].

The medical information included age at the initial procedure (index) date, as well as sex, race, and comorbidities of hypertension, acute kidney injury, diabetes, ischemic heart disease, heart failure, atrial fibrillation, disorders of lipoprotein metabolism disorders, and other dyslipidemias, obesity, history of nicotine dependence, chronic respiratory disease, cirrhosis, alcohol abuse or dependence, and peripheral vascular disease, recorded up to the date of the index date. Our primary outcomes of interest were shunt infection and shunt revision rates. These outcomes were obtained over five years. Chi-square analysis was performed on categorical variables. Comparisons were made between same-day G-tube and shunt versus each cohort. 

## Results

We identified 4,269 patients with a VPS and G-tube. In addition, 179 (4.19%) patients had a VPS and G-tube placed the same day, 509 (11.92%) patients with a prior VPS and G-tube placement within 1-10 days, 814 (19.07%) within 11-30 days, and 897 (21.01%) with placement after one month. Thus, there were 278 (6.51%) patients with prior G-tube and placement of a VPS within 1-10 days, 342 (8.01%) within 11-30 days, and 1,250 (29.28%) with placement after one month. Baseline demographics and characteristics can be seen in Table 1.

**Table 1:** Baseline demographics and population characteristics

ICD-10 Code	Diagnosis	Same day G-tube/shunt, n (%)	Prior shunt, G-tube within 1-10 days, n (%)	Prior shunt, G-tube within 11-30 days, n (%)	Prior shunt, G-tube after 1 month, n (%)	Prior G-tube, shunt within 1-10 days, n (%)	Prior G-tube, shunt within 11-30 days, n (%)	Prior G-tube, shunt after 1 month, n (%)
AI	Age at Index in years	47.16 (100.00)	49.55 (100.00)	51.58 (100.00)	34.39 (100.00)	47.32 (100.00)	45.30 (100.00)	41.59 (100.00)
M	Male	124 (70.06)	342 (67.46)	563 (69.51)	572 (63.69)	168 (60.00)	198 (57.89)	770 (62.19)
F	Female	74 (41.81)	206 (40.63)	326 (40.25)	426 (47.44)	112 (40.00)	144 (42.11)	506 (40.87)
2106-3	White	103 (58.19)	301 (59.37)	484 (59.75)	472 (52.56)	165 (58.93)	189 (55.26)	732 (59.13)
2054-5	Black or African American	35 (19.77)	95 (18.74)	134 (16.54)	162 (18.04)	83 (29.64)	103 (30.12)	266 (21.49)
2131-1	Unknown Race	15 (8.48)	56 (11.05)	91 (11.24)	142 (15.81)	25 (8.93)	35 (10.23)	154 (12.44)
2028-9	Asian	<10 (<5.65)	12 (2.37)	17 (2.09)	18 (2.00)	<10 (<3.57)	<10 (<2.92)	35 (2.83)
I10-I16	Hypertensive Diseases	50 (28.25)	327 (64.49)	575 (70.99)	429 (47.77)	173 (61.79)	195 (57.02)	734 (59.29)
R13	Aphagia and Dysphagia	25 (14.12)	270 (53.25)	428 (52.84)	561 (62.47)	165 (58.93)	172 (50.29)	297 (23.99)
R40	Somnolence, Stupor and Coma	22 (12.43)	222 (43.79)	429 (52.96)	468 (52.12)	145 (51.79)	186 (54.39)	724 (58.48)
F17	Nicotine Dependence	20 (11.29)	127 (25.05)	214 (26.42)	368 (40.98)	64 (22.86)	91 (26.61)	521 (42.08)
N17-N19	Acute Kidney Failure and Chronic Kidney Disease	18 (10.17)	122 (24.06)	194 (23.95)	228 (25.39)	62 (22.14)	72 (21.05)	252 (20.36)
E78	Lipoprotein Metabolism Disorders and Other Dyslipidemia	16 (9.04)	116 (22.88)	216 (26.67)	12 (1.34)	60 (21.43)	74 (21.64)	291 (23.51)
R63	Symptoms and Signs Concerning Food and Fluid Intake	15 (8.48)	108 (21.30)	183 (22.59)	159 (17.71)	56 (20.00)	72 (21.05)	197 (15.91)
I20-I25	Ischemic Heart Diseases	14 (7.91)	87 (17.16)	190 (23.46)	214 (23.83)	56 (20.00)	62 (18.13)	239 (19.31)
I48	Atrial Fibrillation and Flutter	<10 (<5.65)	51 (10.06)	88 (10.86)	119 (13.25)	31 (11.07)	37 (10.82)	103 (8.32)
I50	Heart Failure	<10 (<5.65)	85 (16.77)	149 (18.39)	28 (3.12)	39 (13.93)	40 (11.69)	16 (1.292)
I73	Other Peripheral Vascular Diseases	<10 (<5.65)	<10 (<1.97)	<10 (<1.24)	43 (4.79)	<10 (<3.57)	17 (4.97)	66 (5.33)
E08-E13	Type 2 Diabetes Mellitus	14 (7.91)	87 (17.16)	165 (20.37)	130 (14.48)	51 (18.21)	58 (16.96)	381 (30.78)
J40-J47	Chronic Lower Respiratory Diseases	11 (6.22)	86 (16.96)	150 (18.52)	80 (8.91)	44 (15.71)	59 (17.25)	180 (14.54)
Z87.891	Personal History of Nicotine Dependence	<10 (<5.65)	74 (14.59)	116 (14.32)	212 (23.61)	33 (11.79)	38 (11.11)	272 (21.97)
R53	Malaise and Fatigue	<10 (<5.65)	61 (12.03)	120 (14.82)	92 (10.25)	45 (16.07)	38 (11.11)	272 (21.97)
F10.1	Alcohol Abuse	<10 (<5.65)	39 (7.69)	68 (8.39)	142 (15.81)	27 (9.64)	33 (9.65)	52 (4.20)
F10.2	Alcohol Dependence	<10 (<5.65)	20 (3.95)	43 (5.31)	296 (32.96)	14 (5.00)	19 (5.56)	128 (10.34)
K74	Fibrosis and Cirrhosis of Liver	<10 (<5.65)	18 (3.55)	50 (6.17)	18 (2.00)	<10 (<3.57)	<10 (<2.92)	140 (11.31)

Patients who had same-day G-tube and VPS placement had a VPS infection rate of 10.06% within 5-years, and 14.53% had a VPS revision. In the prior VPS group with subsequent G-tube placement within 1-10 days, 12.18% had VPS infections (p=0.45), and 17.88% had VPS revisions (p=0.30); for those who had subsequent G-tube placement within 11-30 days, VPS infections were seen in 10.57% (p=0.84) of patients, and VPS revisions in 19.41% (p=0.13); G-tube placement after one month or longer of VPS placement resulted in 15.39% of patients having VPS infections (p=0.064) and 17.73% having a VPS revision (p=0.30). In patients with a G-tube with subsequent VPS placement within 1-10 days, VPS infection rates were 8.27% (p=0.52), and VPS revision rates were 14.39% (p=0.97); for VPS placement within 11-30 days of G-tube placement, VPS infections were seen in 10.82% (p=0.79), and VPS revisions were done in 14.33% (p=0.95) of patients; for VPS placement after one month or longer of G-tube placement, VPS infection rate was 11.68% (p=0.52) and shunt revision rate was 16.80% (p=0.44). (Table 2)

**Table 2:** Rates of VPS infection and VPS revision

	Total n	VPS infection n, (%)	VPS revision n, (%)
Same day G-tube/VPS	179	18 (10.06)	26 (14.56)
Prior VPS, G-tube within 1-10 days	509	62 (12.18)	91 (17.88)
Prior VPS, G-tube within 11-30 days	814	86 (10.57)	158 (19.41)
Prior VPS, G-tube after 1 month	897	138 (15.39)	159 (17.73)
Prior G-tube, VPS within 1-10 days	278	23 (8.27)	40 (14.39)
Prior G-tube, VPS within 11-30 days	342	37 (10.82)	49 (14.33)
Prior G-tube, VPS after 1 month	1250	146 (11.68)	210 (16.80)

## Discussion

Our results demonstrate no significant difference in VPS infection rates and VPS revision rates between same-day G-tube and VPS placement versus placement within 1-10 days, 11-30 days, or any other time after one month from one another. While previous studies have shown a VPS infection rate of 0-30%, our study shows an infection rate of 8.27%-15.39% within five years [1-6]. VPS revision rates ranged from 14.33% to 19.41% within five years of follow-up.

In 2020 Tyler et al. published a retrospective analysis looking at G-tube placement and VPS placement within the same hospitalization. They found a VPS infection rate in three out of 45 patients (7%) [5].

In 2017 Oterdoom et al. published a systematic review of VPS and G-tube placement. They found nine relevant studies and overall found VPS infections in 26 out of 208 patients (12.5%). In addition, 137 out of 208 patients had VPS before G-tube placement, with a VPS infection rate of 4.4%; 55 patients had G-tube placement before VPS, with a resulting infection rate of 21.8%; 16 patients had G-tube and VPS placement during the same day, and the infection rate was 50%. The authors concluded that G-tube placement ideally occurs before VPS placement but that having a VPS is not a contraindication to G-tube placement [2].

In 2009, Kim et al. analyzed patients requiring a G-tube both with and without a pre-existing VPS. Of 55 patients, seven (12.7%) had pre-existing shunts. The mean interval between VPS and G-tube placement was 300 days. No patients experienced VPS infections, and the overall complication rate did not differ between the two groups [1]. Cairns et al. in 2009 reported a total of 13 G-tubes placed in 11 patients with prior VPS. One patient had a VPS infection 54 days after G-tube. They also looked at 13 patients with G-tube before VPS placement, and four (30.7%) of these patients had VPS infection. Overall, VPS infection was 20.8%, and the difference between infection rates was not statistically significant (p=0.52). Likewise, patients who had the two procedures performed within 10 days had the highest incidence of infection (30%), with no statistical significance (p=0.67) [6].

Roeder et al. in 2006 examined 55 patients with VPS and G-tube placement. Of 55 patients, seven (12.5%) developed infections. The authors concluded that G-tube placement with VPS is safe and that the order of device placement does not play a significant role [3]. A year before this study, Schulman et al. published a retrospective single-center study of 39 patients with VPS who eventually required a G-tube. The time interval between VPS and G-tube placement was 2 to 564 days. Only two (5%) patients developed meningitis, which was at the 2- and 15-month mark after G-tube placement [4].

A 2021 systematic review of G-tube and VPS placement in the pediatric population by Gerges et al. found four studies involving the timing of VPS and G-tube placement, which reported inconclusive results, with some study patients having no infections with the concomitant placement of both devices, and other studies showing increased risk of shunt infection with prior G-tube placement [11-15].

The major limitation of this study was its retrospective design for data obtention. Furthermore, due to the nature of the database, we were unable to collect patient-level data. Another limitation of this study was the unavailability of radiological images and reports. Also, the diagnostic protocol and tests performed to assess diagnoses were unavailable in the database we utilized/employed. In addition, some misidentification is inevitable in database studies.

## Conclusions

In this large database retrospective study, we approached the inconclusive timing between VPS and G-tube placement related to device-related and device-placement infection rates. Our results conclude that there is no significant difference in VPS infection rates or VPS revision rates between same-day G-tube and VPS placement versus placement within 1-10 days, 11-30 days, or any other time after one month from one another. This suggests that these procedures are safe to perform concurrently and that either procedure may not limit the timing of the other.
